# Roles of the Fc Receptor γ-Chain in Inducing Protective Immune Responses after Heterologous Vaccination against Respiratory Syncytial Virus Infection

**DOI:** 10.3390/vaccines9030232

**Published:** 2021-03-08

**Authors:** Hye Suk Hwang, Young-Tae Lee, Ki-Hye Kim, Ho Seong Seo, Kap Seung Yang, Hoonsung Cho, Sang-Moo Kang

**Affiliations:** 1 Alan G. MacDiarmid Energy Research Institute, Chonnam National University, Gwangju 61186, Korea; hshwang33@chonnam.ac.kr (H.S.H.); ksyang@jnu.ac.kr (K.S.Y.); 2 School of Materials Science & Engineering, Chonnam National University, Gwangju 61186, Korea; 3 Center for Inflammation, Immunity & Infection, Institute for Biomedical Sciences, Georgia State University, Atlanta, GA 30303, USA; leechard75@gmail.com (Y.-T.L.); kihyekim4282@gmail.com (K.-H.K.); 4 Research Division for Radiation Science, Korea Atomic Energy Research Institute, Jeongeup 56212, Korea; hoseongseo@kaeri.re.kr; 5 Department of Polymer Engineering, Graduate School, School of Polymer Science and Engineering, Chonnam National University, Gwangju 61186, Korea

**Keywords:** Fc receptor, RSV F-virus-like particles, cytotoxic T-cells

## Abstract

The roles of the Fc receptor (FcR) in protection or inflammatory disease after respiratory syncytial virus (RSV) vaccination and infection remain unknown. Virus-like particles containing RSV fusion proteins (RSV F-VLPs) induce T-helper type 1 antibody responses and protection against RSV. Heterologous RSV F-VLP prime and formalin-inactivated RSV (FI-RSV) boost vaccination has been reported to be effective in providing protection without inflammatory disease. Here, we investigated whether the FcRγ-chain is important for immune protection by the heterologous F-VLP and FI-RSV vaccination using FcRγ-chain knockout (−/−) mice. RSV F-VLP-primed and FI-RSV-boosted FcRγ −/− mice displayed less protective efficacy, as shown by higher lung viral titers upon RSV challenge, compared to RSV F-VLP-primed and FI-RSV-boosted immunized wild-type mice. RSV F-VLP and FI-RSV immunization induced lower levels of neutralizing activity and interferon-γ-producing CD8 T-cells in the bronchoalveolar lavage cells of FcRγ −/− mice than in those of wild-type mice. In addition, FcRγ −/− mice displayed a trend of enhancing lung histopathology after RSV vaccination and infection. This study suggests that the FcRγ-chain plays an important role in inducing antiviral protection and CD8 T-cell responses in RSV F-VLP prime and FI-RSV boost vaccination after RSV infections.

## 1. Introduction

Respiratory syncytial virus (RSV) is a pathogen that is the leading cause of severe respiratory disease in infants, children, and the elderly. Despite decades of research, there is no licensed RSV vaccine. Formalin-inactivated RSV (FI-RSV) vaccines have failed because of vaccine-enhanced respiratory disease (ERD) in young vaccinee children after exposure to RSV infection during the following epidemic season [1,2]. Virus-like particles (VLPs) are a unique delivery platform for virus structural proteins, mimicking viral morphology without genetic materials, and their advantages lie in their safety, immunogenicity, and antigen stability [3]. VLP vaccine platforms have been reported to avoid ERD in preclinical studies. VLP-based RSV vaccines assemble with the human metapneumovirus (hMPV) matrix protein (M) as the structural scaffold and the RSV fusion glycoprotein (F) in either post-fusion or pre-fusion conformation-induced T-helper type 1 (Th1)-mediated immune responses, which are desirable for avoiding ERD [4]. The chimeric Newcastle disease virus (NDV) RSV VLP vaccine induces a high titer of RSV-neutralizing activity and IgG2a isotype antibodies [5,6] as well as protection without ERD after RSV infection. Priming of mice with live RSV intranasally or inactivated virus intramuscularly determines a specific pattern of T-helper cytokines induced upon subsequent RSV challenge, suggesting an important role of first-exposure vaccine types [7]. Our previous studies have shown that immunization with the RSV VLP vaccine can induce effector memory T-cells [8] and protection against RSV in mice [9,10]. RSV F-VLP vaccination stimulates dendritic cell (DC) activation and maturation and promotes the induction of a Th1 cytokine-producing pulmonary microenvironment and CD8 T-cells that produce interferon (IFN)-γ [8]. It has also been shown that priming of F-VLPs modulates the immune response toward the Th1 pattern and effector CD8 T-cell responses under subsequent ERD-prone FI-RSV vaccination, resulting in the attenuation of ERD by suppressing pulmonary inflammation and eosinophilia [5]. These studies have provided evidence that priming with RSV F-VLPs contributes to the modulation of RSV protective immunity.

RSV F-protein is a major target of CD8 T-cells [11]. The types and balance of T-cell immunity are important for assessing its association with vaccine responses, RSV protection, and immunopathology. A pattern of prior immune response to RSV in very young individuals is likely to play a role in determining the clinical outcome of RSV infection [7,12]. Protection against RSV could depend on the pre-existing pattern of T- and B-cells to be re-stimulated by the vaccine [13]. Priming vaccination with the influenza virus H5 hemagglutinin antigen significantly increased the duration of IFN-γ-producing T-cell responses induced by a heterologous H5 booster vaccination [14].

Previous studies have shown that Fc receptors (FcRs) might contribute to inflammatory RSV disease by accumulating immune complexes during RSV infection [15,16,17] or, independent of IgG immune complexes, by impairing T-cells [18]. Nonetheless, monoclonal antibodies against RSV F- and G-proteins have been shown to provide protection against RSV [19,20,21,22]. RSV-specific antibodies interacting with Fc-gamma receptors might lead to the elimination of virus-IgG immune complexes or the enhancement of viral infections [23,24], which remains to be further investigated. However, the contribution of FcRs to promoting or preventing ERD after RSV vaccination and infection remains unknown. In addition, the possible roles of FcRs in inducing protection against RSV and cellular immunity after RSV vaccination and infection have not been investigated.

VLP vaccines containing RSV-F (F-VLP) have been shown to elicit Th1-type IgG2a isotype antibodies in BALB/c mice [8,25]. In our previous study, we reported that heterologous RSV F-VLP prim and FI-RSV boost vaccination was effective in providing protection while preventing enhanced respiratory and inflammatory disease after RSV infection, compared to the FI-RSV vaccination only [25]. In the present study, we investigated whether FcRγ contributes to inducing protective immunity against RSV via Th1-type immune responses and IFN-γ-producing CD8 T-cells after the heterologous F-VLP prime and FI-RSV boost vaccination, by using a FcRγ knockout (−/−) mouse model. We also determined lung inflammatory histopathology in FcRγ −/− and wild-type mice after RSV vaccination and infection. This study reports that FcRγ is an important host immune component for mediating effective lung viral clearance and inducing effector CD8 T-cell responses by RSV F-VLP priming and subsequent FI-RSV boost vaccination.

## 2. Materials and Methods

### 2.1. Preparation of RSV A2 Virus, RSV F-VLP, and FI-RSV

Influenza virus matrix-protein-derived RSV F-VLP was prepared as previously described [25]. A F-VLP containing RSV A2 fusion (F) protein was produced in insect cells using the recombinant baculovirus expression system as previously described. In brief, Sf9 insect cells were infected with recombinant baculoviruses expressing the influenza virus M1 matrix core protein and RSV A2 fusion (F) protein. Insect cell-culture supernatants containing released RSV F-VLP were harvested, cell debris was removed by low-speed centrifugation, and finally, the RSV F-VLP vaccine was purified using sucrose-gradient ultracentrifugation. For FI-RSV, RSV was inactivated with formalin (1:4000 (vol/vol)) for 72 h at 37 °C. FI-RSV was pelleted by ultracentrifugation (100,800× *g*, 1 h) at 4 °C, resuspended in serum-free medium, and then adsorbed to an alum adjuvant (4 mg/mL, aluminum hydroxide, Sigma Aldrich) for use in the FI-RSV vaccination in this study [25].

### 2.2. Immunization and RSV A2 Virus Challenge

BALB/c wild-type (WT) and FcRγ-deficient mice (FcRγ −/− encoded by Fcer1g on the BALB/c genetic background) were purchased (Taconic Farms, Hudson, NY) and maintained in the animal facility at Georgia State University (GSU). For immunization, 6–8-week-old female BALB/c WT mice (*n* = 5 mice per group) and FcRγ −/− mice (*n* = 5 per group) were intramuscularly immunized with the F-VLP (10 µg) prime and the FI-RSV (2 µg) + alum adjuvant (20 μg) boost for the heterologous (F-VLP/FI-RSV) group at weeks 0 and 4. Naïve control and immunized mice were infected with 0.5 × 10^6^ plaque-forming units (PFU) of RSV A2 under isoflurane anesthesia 4 weeks after boost immunization.

### 2.3. Determination of Antibody Response, RSV Neutralization, and Lung Viral Titration

F-protein-specific antibody titers were determined in prime and boost immune sera using enzyme-linked immunosorbent assay (ELISA) with purified RSV F-protein (100 ng/mL, BEI resources) as a coating antigen [10]. Five hundred times-diluted prime or boost sera were added and incubated for 1.5 h at 37 °C; horseradish-peroxidase-conjugated goat anti-mouse IgG, IgG1, and IgG2a (Southern Biotechnology, Birmingham, AL) were used as secondary antibodies. To determine the RSV-neutralizing activity of immune sera, RSV expressing the red fluorescent monomeric Katushka 2 protein (A2-K-line19F) was used as previously described [25]. Briefly, 500 PFU of live A2-K-line19F RSV and complement-inactivated immune sera at 56 °C were mixed for 30 min, added to the confluent HEp-2 cell monolayer plates, adsorbed for 2 h at 37 °C, and then the antibody–virus mixture was removed. The mean percentages of fluorescence reduction by sera from vaccinated mice and sera from naïve controls were determined and compared [26]. The bronchoalveolar lavage (BAL) fluid (BALF) of individual mice was collected by infusing 1 mL of PBS into the lungs via the trachea using a 25-gauge catheter at day 5 post-challenge. RSV titers were determined in the lung samples [10]. Briefly, serially diluted lung homogenates were added to the confluent HEp-2 cell monolayer plates, adsorbed for 2 h at 37 °C, and then incubated at 37 °C for 3 days. After fixation with 5% formaldehyde, the plate was developed using a mouse anti-RSV F-monoclonal antibody and HRP-conjugated anti-mouse IgG antibody using a 3,3′-diaminobenzidine tetrahydrochloride (DAB) substrate (Invitrogen). The detection limit of the viral titer from the lung samples was 50 PFU.

### 2.4. Cytokine-Expressing T-Cells and Analysis of BAL Cells Phenotype by FACS

For cell phenotype analysis in the mucosal compartment, BAL cells from individual mice (*n* = 5 per group) were prepared at day 5 post-challenge. BAL cells were stimulated with 4 µg/mL RSV peptides [25,27,28], F_92–106_ (ELQLLMQSTPATNNR), for CD8 T-cells for 5 h prior to assessing IFN-γ, TNF-α, or IL-4 cytokine-producing cells. Intracellular cytokines and surface markers for T-cells were stained with antibodies for IFN-γ, IL-4 (eBioscience), TNF-α (BioLegend), CD45, CD3, CD4, CD8, DX5, CD11c, and B220 (BD Biosciences). Stained cells were acquired on a FACSCanto flow cytometer (BD Biosciences) and analyzed using FlowJo software (Tree Star, Inc., Ashland, OR, USA).

### 2.5. Histopathology

Lung tissues collected from mice at 5 days after RSV challenge were fixed with 10% neutral buffered formalin. Lung tissue histology was performed by staining with hematoxylin and eosin (H&E), periodic acid–Schiff (PAS), and hematoxylin and congo red (H&CR) and analyzed under light microscopy [10,28,29]. The sections around the airways were scored for the degree of inflammation according to the following scale: 0 (none), 1 (mild), 2 (moderate), and 3 (severe) [30]. The areas of epithelium and PAS-positive areas within the airway epithelium were annotated using the magnetic lasso tool of Adobe Photoshop CS5.1 software. The degrees of pulmonary eosinophilia were identified by H&CR staining to enumerate eosinophils and were expressed as the number of eosinophils present per 400X field.

### 2.6. Statistical Analysis

All results are represented as the mean ± standard error of the mean (SEM). Significant differences among treatments were investigated by one-way analysis of variance (ANOVA), using Tukey’s or Dunnett’s multiple-comparison test, or two-way ANOVA with the Bonferroni post-test in GraphPad Prism version 5 (GraphPad Software, Inc., San Diego, CA, USA). *p*-Values ≤ 0.05 were considered statistically significant.

## 3. Results

### 3.1. Lower Neutralizing Titers in FcRγ −/− Mice after RSV F-VLP/FI-RSV Vaccination

**Figure 1:**
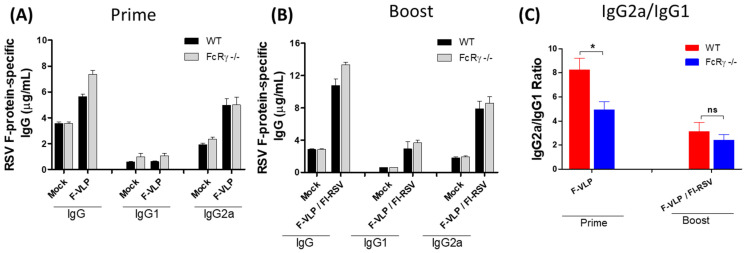
Antibody responses specific for respiratory syncytial virus (RSV)-F proteins in the serum of wild-type (WT) and Fc-receptor-γ-deficient (FcRγ −/−) mice. FcRγ −/− mice (*n* = 5 per group) and WT BALB/c mice (*n* = 5) were intramuscularly (i.m.) immunized at week 0 (virus-like particle vaccine containing RSV-F protein (F-VLP) prime) and 4 (formalin-inactivated RSV (FI-RSV) boost). (**A**) F-specific IgG and isotypes after the prime. (**B**) F-specific IgG and isotypes after the boost. (**C**) Ratios of IgG2a to IgG1 isotype antibodies. F-VLP: RSV F-VLP (10 μg). F-VLP/FI-RSV: heterologous F-VLP prime and FI-RSV (2 μg + alum adjuvant 20 μg) boost. Statistical analysis is as follows. Results are presented as mean ± SEM. * *p* < 0.05. n.s.: not significant.

To evaluate the possible roles of FcRγ in inducing protective immune responses to RSV, the groups of WT and FcRγ −/− mice were intramuscularly immunized via a heterologous F-VLP prime and FI-RSV boost regimen. As shown in Figure 1, immunization of FcRγ −/− mice induced similar or slightly higher levels of RSV F-specific antibodies compared to those in WT mice at 3 weeks after the prime and boost vaccination (Figure 1A,B). Similarly, the F-VLP prime immunization of WT and FcRγ −/− mice elicited an IgG2a isotype-dominant response (Figure 1A,C) although IgG2a/IgG1 isotype ratios were slightly decreased in FcRγ −/− mice (Figure 1C). These results suggest that FcRγ −/− mice do not have a defect in inducing IgG antibody responses.

**Figure 2:**
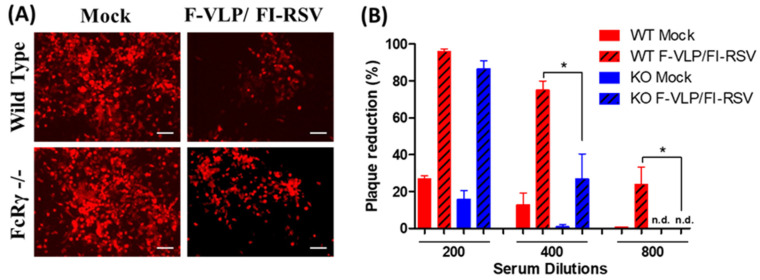
RSV-neutralizing titers of serum samples collected at 2 weeks after the boost. Diluted sera incubated with RSV A2-K-line19F (500 PFU) were added to HEp-2 cell monolayers to determine the reduction (%) in fluorescence intensity. (**A**) Fluorescence images of RSV-neutralizing activity in mock control or F-VLP/FI-RSV-immunized sera of WT and FcRγ −/− mice. (**B**) Data are presented as mean fluorescence reduction percentages ±SEM (*n* = 5 per group). * *p* < 0.05.

To evaluate the possible roles of FcRγ in inducing virus-neutralizing antibodies by the RSV F-VLP prime and FI-RSV boost, serum samples collected at 2 weeks after the boost were tested for neutralizing activity. The representative fluorescence microscope images at 400× serum dilution shown in Figure 2A,B show that both WT and FcRγ −/− mice with F-VLP/FI-RSV vaccination induced similar neutralizing activity at low 200x dilution of sera. At 1:400 dilution, antisera of FcRγ −/− mice showed a 26% reduction in fluorescence intensity versus a 75% reduction within antisera from WT mice after F-VLP/FI-RSV vaccination. At 1:800 dilution, antisera of the RSV F-VLP/FI-RSV immunization in WT mice showed a 23% reduction in fluorescence intensity. However, no fluorescence reduction was observed in antisera from FcRγ −/− mice. These results indicate that FcRγ plays a role in inducing neutralizing antibodies after heterologous RSV F-VLP/FI-RSV vaccination.

### 3.2. FcRγ Contributes to the Effective Clearance of Lung Viral Loads by F-VLP/FI-RSV Vaccination

**Figure 3:**
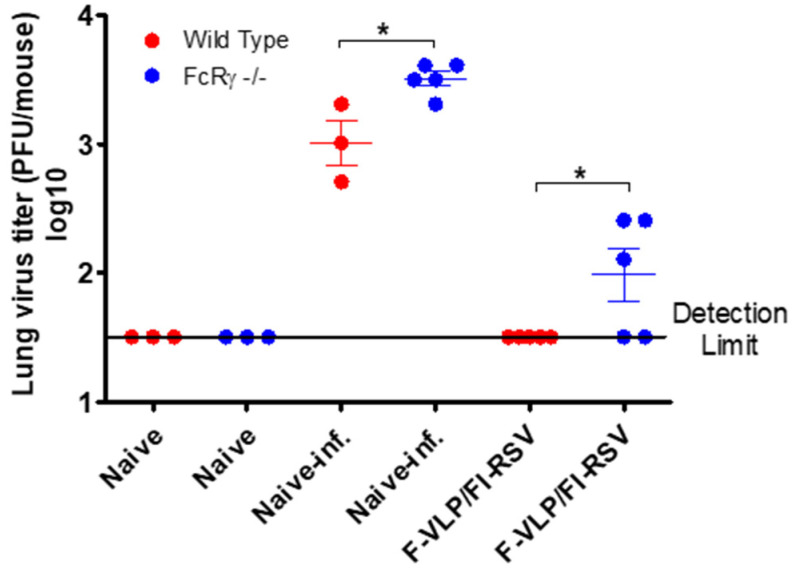
FcRγ −/− mice were less effective in clearing lung viral titers than WT mice after RSV infection. F-VLP/FI-RSV-immunized mice were challenged with RSV (0.5 × 10^6^ PFU/mouse) at 4 weeks after boost vaccination. Lung viral loads in each mouse (PFU/mouse lung) were determined at 5 days after infection by an immuno-plaque assay. Naïve inf.: unimmunized naïve mice after RSV. *n* = 3 for naïve or naïve-infection of WT, *n* = 5 for the rest of the groups. Statistical significance: * *p* < 0.05.

Naïve and immunized mice were challenged with live RSV A2 virus (0.5 × 10^6^ PFU/mouse) 4 weeks after the boost, and lung viral loads at day 5 post-challenge were determined. FcRγ −/− naïve mice exhibited higher lung viral titers of RSV (3.5 log10) than WT mice (Figure 3). Consistent with the results of neutralizing activity, F-VLP/FI-RSV-vaccinated WT mice controlled RSV lung viral loads more effectively to a level below the detection limit, compared to FcRγ −/− mice which displayed substantial levels of lung viral loads (Figure 3). These results indicate that FcRγ plays a role in lung viral clearance after RSV infection in F-VLP/FI-RSV-immune mice.

### 3.3. FcRγ Is Important for Inducing RSV F-Specific CD8 T-Cell Responses

**Figure 4:**
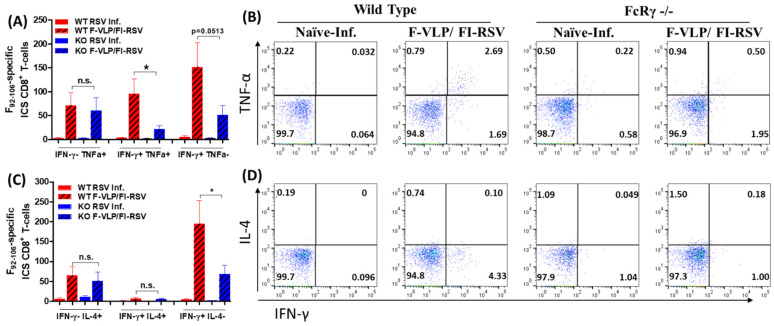
RSV F-specific CD8 T-cells secreting IL-4, TNF-α, and IFN-γ. BAL cells collected from mice (*n* = 5 per group) at day 5 post-challenge were stimulated with the RSV-F_92–106_ CD8 peptide epitope. (**A**) The numbers and (**B**) populations of CD8+ T-cells secreting IFN-γ (TNF-α-IFN-γ+) or TNF-α (TNF-α+IFN-γ-) out of the total BAL cells collected from individual mice. (**C**) The numbers and (**D**) populations of CD8+ T-cells secreting IL-4 (IL-4+IFN-γ-) or IFN-γ (IL-4 -IFN-γ+) out of the total BAL cells collected from individual mice. Results are presented as mean ± SEM. * *p* < 0.05, comparing indicated groups. n.s.: not significant.

In addition to the potential necessity of neutralizing antibodies against live RSV infection, T-cell immune responses could contribute to the protection against RSV infection [23]. Therefore, we determined the induction of RSV F-specific CD8 T-cells by intracellular cytokine staining of BAL cells at day 5 post-challenge (Figure 4). IFN-γ-TNF-α+ CD8 T-cells and IFN-γ-IL-4+ CD8 T-cells were detected at similarly low levels in BALF from both WT and FcRγ −/− mice with the F-VLP/FI-RSV vaccination after RSV challenge. Double positive IFN-γ+TNF-α+ CD8 T-cells were induced at higher levels in WT BALF than in FcRγ −/− BALF samples (Figure 4A,B). Double-positive IFN-γ+IL-4+ CD8 T-cells were not detected in the non-immunized groups. IFN-γ+TNF-α- and IFN-γ+IL4- CD8 T-cells were observed at higher levels in BAL cells from WT mice than those from FcRγ −/− mice with F-VLP/FI-RSV immunization (Figure 4A–D). Therefore, these results suggest that FcRγ plays a pivotal role in inducing effector CD8 T-cell responses, which might contribute to protection. 

### 3.4. FcRγ Plays a Role in Recruiting CD8 T-Cells and NK Cells into Airway BALF in RSV F-VLP/FI-RSV-Vaccinated Mice after RSV Infection

**Figure 5:**
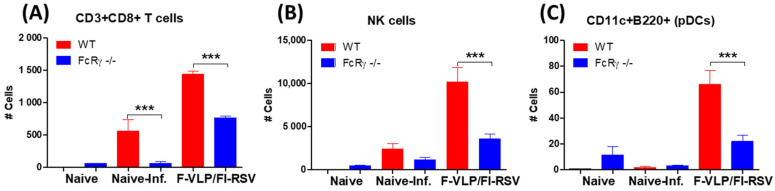
The number of CD8 T-cells, NK cells and plasmacytoid dendritic cells (pDCs) out of the total BAL cells collected per mouse (*n* = 5 per group). In the bronchoalveolar lavage fluid (BALF) after challenge, BAL cells were harvested, stained with CD45, CD3, CD8, DX5, CD11c, and B220, and analyzed using flow cytometry. (**A**) CD3+CD8+ T-cells, (**B**) DX5+ NK cells, and (**C**) CD11c+B220+ pDCs. Results are presented as mean ± SEM. *** *p* < 0.001.

VLP priming can induce cytotoxic T-cell responses via direct- and cross-presentation by dendritic cells (DCs) [31,32]. NK cells recognize particulate antibody–antigen immune complexes on infected cells that bind to receptors for the Fc portion of Ig (FcR) expressed on these cells and trigger antibody-dependent cell-mediated cytotoxicity (ADCC) [23,33]. Furthermore, NK cells are important innate immune cells in the early response to and control of viral infections [34], and their activity is tightly regulated, for example, via their interaction with antigen-specific antibodies [35]. The CD8 T-cells, NK cells, and dendritic cells (DCs) phenotype of BAL cells were analyzed to determine whether the deletion of FcRγ would affect the recruitment of CD8 T-cells, NK cells, and dendritic cells (DCs) into the airways after RSV infection in F-VLP/FI-RSV-immunized mice. The WT mice with immunization recruited higher levels of CD3+CD8+ T-cells, NK cells, and CD11c+B220+ DCs after RSV infection compared to FcRγ −/− mice (Figure 5A–C). Therefore, these results provide evidence that FcRγ plays a role in recruiting CD3+CD8+ T-cells, NK cells, and CD11c+B220+ DCs into the airway respiratory sites in F-VLP/FI-RSV-vaccinated mice after RSV infection.

### 3.5. FcRγ Plays a Role in Diminishing Histopathology in F-VLP/FI-RSV-Vaccinated Mice after Challenge

**Figure 6:**
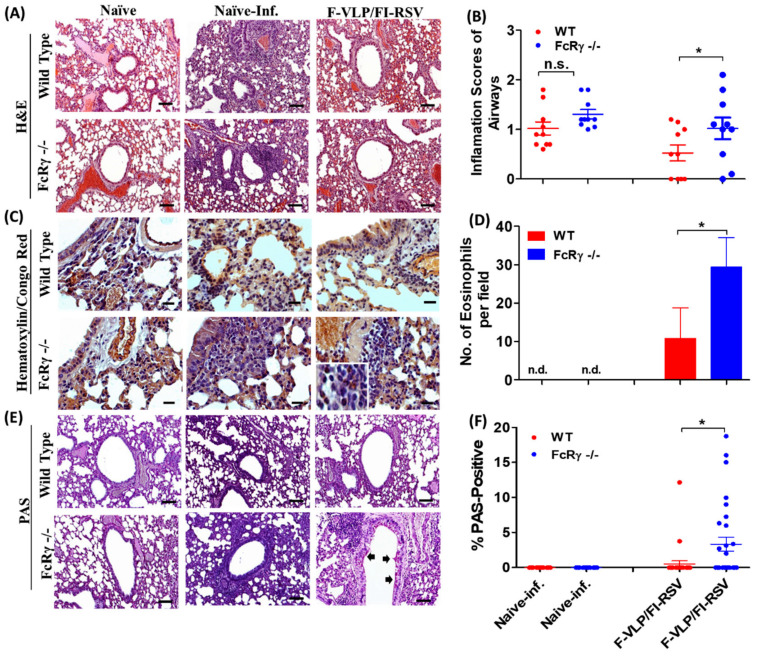
Lung histopathology, eosinophilic infiltration, and mucus production of lungs in WT and FcRγ −/− mice after RSV challenge (*n* = 5 per group). (**A**) H&E staining of lung tissues. (**B**) H&E-stained tissue sections from each mouse were scored for inflammation on a scale of 0–3. (**C**) Hematoxylin/Congo red (H&CR) staining to determine pulmonary eosinophilia and (**D**) number of eosinophils per 40× field in two different regions of each mouse. (**E**) Periodic acid–Schiff (PAS) staining to determine mucus production. (**F**) PAS-positive areas in airways (10 individual airways in each mouse) were quantified and represented as a percentage. Results are presented as mean ± SEM. * *p* < 0.05. n.d.: not detected.

To assess differences in lung histopathology between WT and FcRγ −/− mice after RSV infection, histology tissues infiltrated with eosinophils and mucus-producing cells in the airways were visualized with H&E, H&CR, and periodic acid–Schiff (PAS) staining. In the groups vaccinated with the F-VLP prime and FI-RSV boost regimens, FcRγ −/− mice displayed higher levels of H&E-stained inflammation (Figure 6A,B), H&CR staining of eosinophils (Figure 6C,D), and PAS-positive histopathology (Figure 6E,F) than WT vaccinated mice (Figure 6). WT mice that were immunized with F-VLP/FI-RSV showed slightly less pulmonary eosinophil infiltration and PAS-positive mucus production, compared to FcRγ −/− mice vaccinated with F-VLP/FI-RSV (Figure 6). These results suggest a role of FcRγ in attenuating histopathology in vaccinated mice after RSV infection.

## 4. Discussion

VLPs contain a high density of repetitive effective antigenic epitopes on their surfaces. DCs and B-cells function as antigen-presenting cells (APCs); therefore, APCs directly take up VLPs and present peptide-form antigens to activate T-cells [36]. Cross-linking of B-cell receptors by VLPs can be strong enough to prime B-cells and induce the production of antibodies even without the help of CD4+ T-cells [37]. Owing to their distinct multimeric-nature conformational advantage, VLP-based vaccines are capable of inducing both humoral and cellular immune responses, and adjuvant co-administration is not needed in most VLP vaccinations [38].

A previous study has shown that priming with RSV F-VLP shifts the immune response toward the Th1 pattern and effector CD8 T-cell responses under subsequent exposure to the ERD-prone FI-RSV vaccine against RSV infection [25]. However, Th1-type-biased RSV F-VLP immune mechanisms are not well understood. In this study, we attempted to better understand the roles and contributions of FcRγ by comparing immune responses and protection in WT and FcRγ −/− mice immunized with F-VLP/FI-RSV. Anti-RSV F IgG antibody and IgG isotype distribution in sera were found to be similar in WT and FcRγ −/− mice after F-VLP priming or F-VLP/FI-RSV vaccination. Therefore, this study suggests that FcRγ is not required for inducing vaccine-specific binding of IgG antibodies after F-VLP/FI-RSV. However, the F-VLP/FI-RSV WT mice induced higher levels of RSV-neutralizing activity in sera, compared to those in F-VLP/FI-RSV FcRγ −/− mice, providing evidence for a role of FcRγ in inducing protective neutralizing antibodies after vaccination against RSV. This role of FcRγ is further supported by the effective clearance of lung RSV loads in WT F-VLP/FI-RSV mice or the lower lung RSV titers in WT naïve mice after RSV infection, compared to those in FcRγ −/− mice.

DCs are known to be professional APCs, highly effective in cross-presenting non-replicating VLP antigens to CD8 T-cells to prime cytotoxic CD8 T-cell responses [31]. VLP-specific antibodies might contribute to more effective uptake via Fc and FcR interactions by DCs, promoting the cross-presentation of antigens to CD8 T-cells. Consistent, higher levels of effector CD8 T-cell responses were induced in WT F-VLP/FI-RSV mice, the levels of which were diminished in FcRγ −/− F-VLP/FI-RSV mice. Hemagglutinin-containing VLPs can prime influenza virus-specific CD8 T-cell responses that contribute to protection from influenza infection [39]. Interestingly, we found that FcRγ −/− naïve mice showed a defect in controlling lung RSV titers compared to WT naïve mice after RSV infection. FcR-mediated viral clearance appears to contribute to controlling viruses in naïve mice after infection. Particularly, FcRγ has been reported to play a critical role in providing broad cross-protection against conserved influenza epitopes in the presence of non-neutralizing antibodies [40,41,42,43]. It is possible that FcRs play a role in inducing CD8 T-cells and mediating viral clearance, both of which contribute to protection.

The Sf9 insect cell-system-based RSV F- or G-VLP has been shown to modulate the immune response to suppress Th2 immune-mediated pulmonary histopathology and eosinophilia [25]. In a subset of individuals, the bacterially produced recombinant VLP-based influenza vaccine shifted pre-vaccination-induced type-2 cytokines such as IL-5 and IL-13 to a post-vaccination type-1 cytokine signature characterized by IFN-γ [44]. In BALB/c mice, IgG2a responses reflect the establishment of Th1-type immune responses for viral infections [45]. We found that levels of IgG2a/IgG1 responses were significantly increased in BALB/c mice after the F-VLP prime vaccination but less so in FcRγ −/− mice. IgG2a antibodies are reported to have higher affinity for the activating receptors FcγRI, -III, and -IV, whereas mouse IgG1 only binds significantly to FcγRIII [46,47].

In this study, we found that FcRγ plays a role in mediating CD8 T-cell cytotoxic immune responses in the local compartment. In the case of SARS-CoV-2 infection, FcRγ engagement on dendritic cells could help stimulate cytotoxic antiviral CD8 T-cell responses, which are often suppressed as a result of excessive viral replication and uncontrolled recruitment of monocytes, leading to Th2-cell-biased airway inflammation and acute lung damage [48,49]. In addition to enhanced VLP antigen uptake and presentation, immune complex endocytosis through activation of type I FcRs is also accompanied by cellular activation and the induction of pro-inflammatory chemokine and cytokine expression, which in turn influence the differentiation, effector activities, and survival of APCs [50,51,52]. IgG antibodies are not required for FcγRIIB-mediated control of CD8 T-cell immunity, and a cell-intrinsic inhibitory function of FcγRIIB directly limited CD8 T-cell responses [53].

A previous study reported the contribution of accumulated immune complexes to enhanced RSV disease [15]. Antibody-dependent enhancement of microbial infection after ligation of macrophage FcRγ via infectious immune complexes has also been reported [16,23]. In contrast, F-VLP/FI-RSV-vaccinated FcRγ −/− mice displayed a trend of increasing histopathology rather than attenuating inflammation after RSV infection. Therefore, this study provides evidence that FcRγ is an important host immune component in clearing lung viral loads and attenuating immune histopathology, at least in a BALB/c mouse model, after F-VLP/FI-RSV vaccination and RSV infection. Collectively, we have shown that FcRγ contributed to inducing RSV F-specific CD8 T-cells and neutralizing antibodies as well as providing effective viral clearance and protection against RSV. In future studies, it is important to determine the types of Fc receptors that mediate IFN-γ-producing CD8 T-cell responses. Further studies are also required to understand and utilize Fc-mediated antibody effector functions for the development of safe and effective vaccines and antibody therapies against RSV.

## 5. Conclusions

The present study demonstrated that the FcRγ-chain plays an important role in inducing antiviral protection and CD8 T-cell responses in RSV F-VLP prime and FI-RSV boost vaccination after RSV infections. Collectively, our data provide important information for FcRγ contributed to inducing RSV F-specific CD8 T-cells and neutralizing antibodies as well as providing effective viral clearance and protection against RSV.

## Data Availability

The data that support the findings of this study are available on request from the corresponding author.
